# Reply to Chen et al. Improvements in Outcomes and Expanding Indications for the Commando Procedure. Comment on “Giambuzzi et al. Surgical Aortic Mitral Curtain Replacement: Systematic Review and Metanalysis of Early and Long-Term Results. *J. Clin. Med.* 2021, *10*, 3163”

**DOI:** 10.3390/jcm11061601

**Published:** 2022-03-14

**Authors:** Ilaria Giambuzzi, Giorgia Bonalumi, Michele Di Mauro, Francesco Alamanni, Marco Zanobini

**Affiliations:** 1 IRCCS Centro Cardiologico Monzino, Department of Cardiovascular Surgery, 20100 Milan, Italy; giorgia.bonalumi@ccfm.it (G.B.); marco.zanobini@ccfm.it (M.Z.); 2 DISCCO Department, University of Milan, 20100 Milan, Italy; francesco.alamanni@unimi.it; 3 Heart and Vascular Centre, Cardio-Thoracic Surgery Unit, Maastricht University Medical Centre (MUMC), Cardiovascular Research Institute Maastricht (CARIM), 6221 Maastricht, The Netherlands; mdimauro1973@gmail.com

We would like to thank Lin Chen et al. [1] for commenting on our manuscript on the Commando surgery [2]. 

At this time, the Commando procedure is still rarely performed and few centers, as Lin Chen et al. [1] pointed out, handle it.

We excluded from our analysis the paper from Davierwala, P.M. et al. [3] as the same authors published another article [4] only a few years later with 127 patients who solely underwent the Commando operation. 

Regarding the other studies with updated populations, data on such an operation are scarce; therefore, we also included patients who underwent the Hemi-Commando procedure, even if, as Lin Chen et al. [1] correctly pointed out, it is a different operation [5,6]. The other studies [7,8] were included because of the long timespan between the two studies and to further underline the improvement of the results over the years. 

We believe that the choice between the Commando and the Hemi-Commando operations can be done by experienced surgeons in high-volume centers because currently there are still a lack of definitive decisions on the relatively few patients who undergo such an aggressive surgery. Therefore, we hope that future research will also focus on improving the pre-operative management of these patients, trying, whenever possible, to avoid a progression of the endocarditic process and to refer patients with intervalvular fibrous body disease to experienced centers with endocarditis teams.

Nevertheless, the Hemi-Commando represents the best surgical choice for patients with a less extensive disease and who might have better long-term results not because of the surgical technique but because of the less aggressive pathological features involving the mitral valve. Future studies with long term results will help to further clarify this point.

Naturally, there are still few data to draw definite conclusions from, and, as Lin Chen et al. say, it is plausible that in the next few years the performance of the Commando and Hemi-Commando procedures in an elective setting (massive calcification, REDO surgery) might improve outcomes and expertise. Furthermore, they are both procedures that should be performed only in high-volume centers by surgeons with extensive experience.
